# Management of Rhino-Orbital-Cerebral Mucormycosis Infection: Anesthetic Challenges

**DOI:** 10.22034/cmm.2025.345516.1743

**Published:** 2026-02-01

**Authors:** Vali Imantalab, Mohammad Dodangeh Balakhani, Iman Haghani, Nazanin Rahmanian, Erfan Vakili, Reihane Einollahzadeh, Hamid Badali, Nooshin Ahmadvand, Gelareh Biazar

**Affiliations:** 1 Anesthesiology Research Center, Department of Anesthesiology, Alzahra Hospital, Guilan University of Medical Sciences, Rasht, Iran; 2 Department of Pulmonology, Faculty of Medicine, Guilan University of Medical Sciences, Rasht, Iran; 3 Invasive Fungi Research Center, Communicable Diseases Institute, Mazandaran University of Medical Sciences, Sari, Iran; 4 Department of Medical Mycology, School of Medicine, Mazandaran University of Medical Sciences, Sari, Iran; 5 Department of Molecular Microbiology and Immunology, South Texas Center for Emerging Infectious Diseases, The University of Texas at San Antonio, San Antonio, Texas, USA; 6 Department of Infectious Disease, Faculty of Medicine, Guilan University of Medical Sciences, Rasht, Iran

**Keywords:** Mucormycosis, Rhino-Orbital-Cerebral, Immunocompromised hosts, Anesthetic management

## Abstract

Despite the application of surgery and antifungal therapy, rhino-orbital-cerebral mucormycosis is a severe and life-threatening fungal infection with high morbidity and mortality rates, particularly in immunocompromised patients. This review aimed to focus on the current evidence on perioperative anesthetic management of rhino-orbital-cerebral mucormycosis (ROCM), emphasizing the unique challenges posed by its aggressive, angioinvasive nature. Key issues included difficult airway management due to fungal debris and supraglottic edema, hemodynamic instability, renal dysfunction from nephrotoxic antifungal therapy (e.g., amphotericin B), and the critical need for glycemic control in diabetic patients. Case studies have illustrated the complexities of managing comorbidities, such as post-COVID-19 sequelae, uncontrolled diabetes, and immunosuppression.

Preoperative optimization, judicious management of fluids and electrolytes, and the use of video laryngoscopy for difficult intubation are highly recommended. By addressing these challenges, this article provided a practical framework for anesthesiologists to enhance perioperative care in patients with ROCM, ultimately improving survival and reducing complications. Awareness of these procedures may help clinicians choose the most effective treatment regimens. However, further studies are warranted to correlate these findings with clinical outcomes.

## Introduction

Mucormycosis is an aggressive, destructive, life-threatening, and angio-invasive fungal infection due to the order Mucorales (Mucor, *Rhizopus*, *Lichtheimia*, *Cunninghamella*, and *Apophysomyces* species) [1-3]. It is associated with poor clinical outcomes despite surgical intervention and antifungal therapy [4]. Mucormycosis, a mucoralean fungal infection, first proliferates in the paranasal sinuses and may subsequently invade the orbital region or intracranial spaces, particularly in immunocompromised individuals, including those with uncontrolled diabetes, neutropenic patients, transplant recipients, burn and trauma victims, and recently in patients with COVID-19 infections [2, 5, 6]. This life-threatening disease is characterized by rapid tissue invasion, vascular thrombosis, and necrosis, leading to high morbidity and mortality rates [7]. The global mortality rate associated with mucormycosis has been reported to be 46% [8]. The most common clinical manifestations are rhino-orbital-cerebral, pulmonary, and disseminated forms. However, rhino-orbital-cerebral mucormycosis (ROCM) is particularly challenging due to its proximity to critical anatomical structures [9]. Management of mucormycosis relies on early diagnosis, aggressive surgical debridement, and systemic antifungal therapy, typically involving liposomal amphotericin B [10]. However, the perioperative period presents unique challenges for anesthesiologists, consisting of complex airway management due to fungal debris and supraglottic edema, hemodynamic instability, and renal dysfunction secondary to drug-induced nephrotoxicity [11, 12]. Additionally, underlying comorbidities, such as uncontrolled diabetes mellitus, hematologic malignancies, and immunosuppression, further complicate anesthetic care. A meta-analysis of case reports identified diabetes mellitus as the most prevalent comorbidity [13], present in 40% of analyzed cases [14]. Consequently, perioperative glycemic control can be challenging, often necessitating interventions, such as glucose-insulin buffered solutions, continuous insulin infusions, or intermittent insulin bolus therapy. Anesthetic considerations must address preoperative optimization, intraoperative stability, and postoperative monitoring to reduce the risks. Difficult intubation, electrolyte imbalances, and the need for invasive hemodynamic monitoring are common concerns [15]. Furthermore, the interplay between mucormycosis and organ dysfunction, particularly renal and pulmonary systems, demands tailored anesthetic strategies to ensure patient safety [16]. This review highlights the current evidence on the anesthetic management of mucormycosis, emphasizing perioperative challenges, multidisciplinary approaches, and evidence-based interventions to improve outcomes in high-risk patient populations (Figure 1).

**Figure 1:**
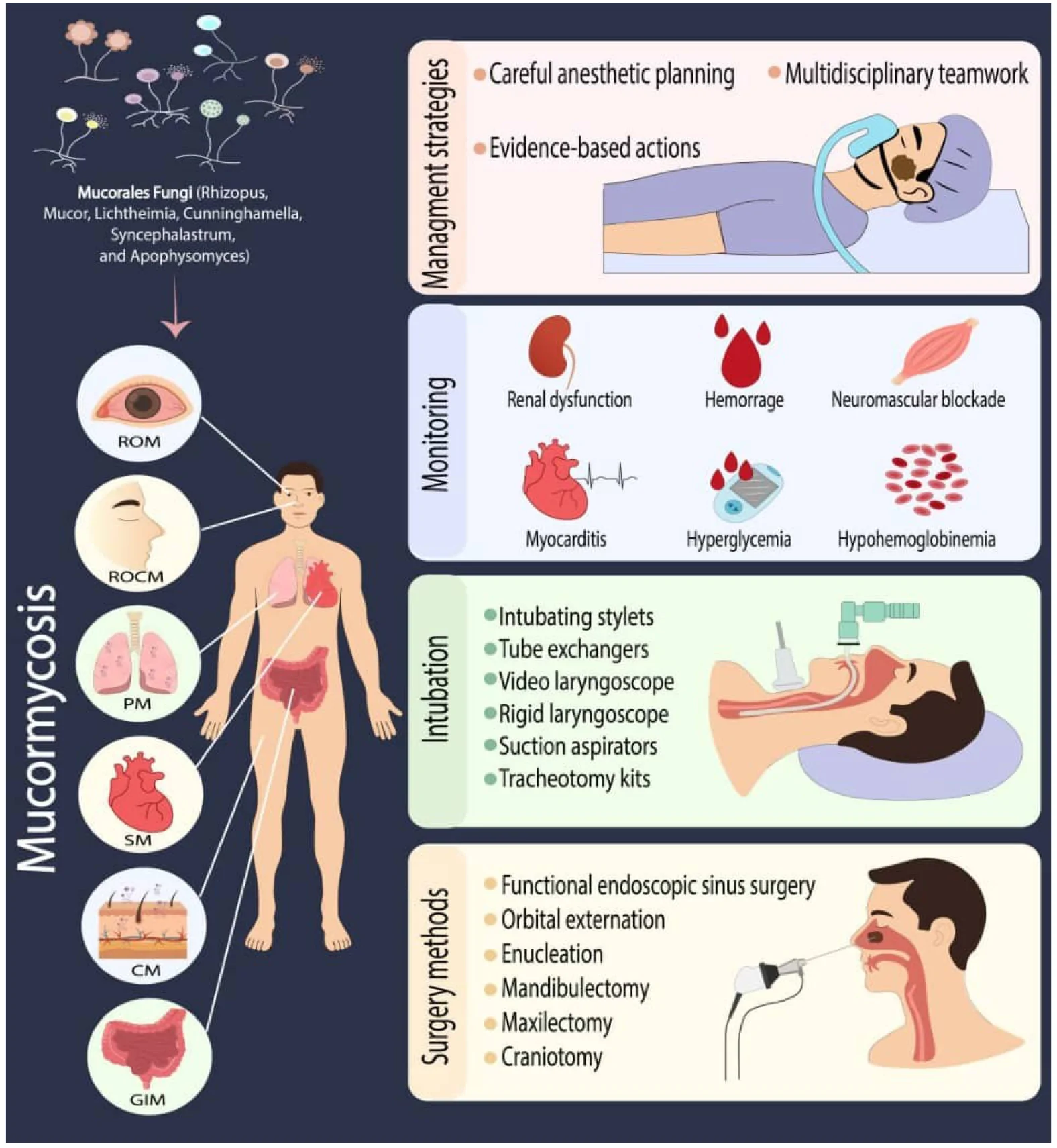
Anesthetic challenges in the management of rhino-orbital cerebral mucormycosis.

## Mucormycosis

### Types of mucormycosis in high-risk patients

Mucormycosis presents in various clinical forms, with rhino-cerebral involvement being the most common, typically affecting the sinuses, nasal passages, and brain, especially in immunocompromised hosts or patients with poorly controlled diabetes. Pulmonary forms are frequently observed in cancer patients, hematopoietic stem cell transplant recipients, or those with severe neutropenia. Gastrointestinal involvement, though rare, is often severe and occurs in malnourished individuals or those with underlying digestive disorders. Disseminated mucormycosis arises from hematogenous spread to vital organs, such as the brain, heart, or spleen, and carries a grave prognosis. Renal involvement is uncommon and generally affects intravenous drug users or severely immunocompromised hosts. Cutaneous forms result from direct inoculation through trauma, burns, surgical wounds, and tattooing [17, 18].

### Clinical manifestations of rhino-orbital-cerebral mucormycosis

Rhino-orbital-cerebral mucormycosis is characterized by rapidly evolving symptoms due to its aggressive nature. Initial signs include unilateral nasal obstruction, bloody or purulent discharge, and localized facial pain. A black necrotic eschar on the nasal mucosa is distinctive, resulting from fungal invasion and tissue death. As the infection progresses to the orbit, patients may experience periorbital edema, proptosis, and chemosis, with ophthalmoplegia and vision loss indicating serious complications. Orbital cellulitis may present similar, but typically involves a more rapid deterioration, distinguishing it from ROCM.

Cerebral extension can present with altered mental status, hemiparesis, seizures, or coma, indicating conditions, such as cavernous sinus thrombosis or meningitis. Horner’s syndrome may arise from stellate ganglion involvement. Advanced cases can lead to life-threatening epistaxis or hemorrhagic stroke due to vascular erosion. Systemic symptoms, including fever and hypotension, often suggest sepsis. Pain disproportionate to clinical findings and rapid progression are key indicators of ROCM, compared to other fungal infections. Mucormycosis is characterized by its angio-invasive nature, potentially leading to hemoptysis and significant airway obstruction, particularly in diabetic patients [4].

### Anesthetic management of rhino-orbito-cerebral mucormycosis

Patients diagnosed with mucormycosis, especially in cases involving ROCM, may encounter difficulties during mask ventilation and endotracheal intubation owing to fungal-induced epiglottitis and edema in the supraglottic region. Therefore, we recommend that anesthesiologists should confirm that the operating theatre is equipped with essential airway management tools, including appropriately sized facemasks, intubating stylets, tube exchangers or gum elastic bougies, laryngeal mask airways, video laryngoscopes, rigid laryngoscope blades of various designs and sizes, fiberoptic-guided intubation devices, suction aspirators, and emergency tracheotomy kits, to manage unforeseen challenging airway scenarios [19] (Figure 1).

In a retrospective study, the presence of widespread fungal debris in the oropharyngeal region of the patient was reported, which, along with supraglottic oedema, prevented successful endotracheal intubation via direct laryngoscopy, necessitating the use of video laryngoscopy instead.

In another study, a patient with mucormycosis was assessed, and a tracheotomy was required since supraglottic edema prevented endotracheal intubation. Notably, the same patient had undergone uneventful intubation two months earlier during abdominal surgery [20]. Remarkably, blood transfusions, fluid resuscitation, and intraoperative hemodynamic support may be necessary for certain patients, often requiring central venous access before, during, or after surgery, as well as during extended intensive care unit (ICU) stays. However, in cases of rhino-orbital-cerebral involvement, the internal jugular vein should be avoided for central venous cannulation due to its proximity to the infected region. Additionally, thrombocytopenia-related pulmonary hematoma poses a risk of postoperative airway compromise, further supporting the need for alternative venous access sites. On the other hand, preventing hypotensive episodes and maintaining hemodynamic stability during the perioperative phase are crucial for ensuring sufficient renal function. Furthermore, ongoing arterial blood gas monitoring, along with intermittent biochemical assessments, is crucial for anaesthetic management, particularly in the evaluation of metabolic status, fluid-electrolyte balance, and coagulopathy [21]. Karaaslan (2019) evaluated five patients with mucormycosis and reported that they developed severe hypotension, requiring positive inotropic support [11]. Given the heightened risk of hemodynamic instability and fluid-electrolyte imbalances in patients with mucormycosis, continuous invasive arterial pressure monitoring and arterial blood gas analysis are essential. Mucormycosis, a vaso-occlusive infection, often leads to tissue necrosis and ischemia due to vascular compromise [22]. Likewise, vascular thrombosis impedes systemic drug delivery to affected tissues, further complicating treatment efficacy.

### Therapy in the anesthetic management of rhino-orbito-cerebral mucormycosis

Optimal choice of intravenous and inhaled anesthetics in patients with active fungal infections, such as mucormycosis, is not well established. Some volatile anesthetics have shown antifungal and antibacterial properties *in vitro* studies [23-25]. Although these agents can inhibit fungal cell growth *in vitro*, their effectiveness *in vivo* needs further verification.

A preliminary study performed by Barodka et al. found that isoflurane inhibited the growth of *Candida albicans* [24, 26]. Antifungal treatment should be initiated using amphotericin B, posaconazole, or isavuconazole; however, caution is advised when administering posaconazole to patients with prolonged QTc intervals [27]. In a study, retrobulbar injection of amphotericin B demonstrated improvement in orbital involvement among patients with ROCM [28]. Administration of systemic amphotericin B for mucormycosis therapy is associated with significant nephrotoxicity and additional adverse effects, such as hypokalemia, hypomagnesemia, fever, chills, dyspnea, hypotension, and thrombophlebitis during infusion [29]. Furthermore, renal impairment may occur in over 80% of patients, with approximately 15% requiring hemodialysis during treatment. Additionally, insulin infusions used in diabetes management may exacerbate hypokalemia Consequently, it is essential to correct potassium levels both before and during surgery, administer fluid resuscitation under central venous pressure monitoring, and provide intravenous paracetamol for fever control. Hypokalemia and hypomagnesemia can result in delayed recovery from neuromuscular blocking agents in surgical anaesthesia and increased susceptibility to cardiac arrhythmias; therefore, regular serum electrolyte monitoring is essential [30]. Moreover, chronic diabetes can result in diabetic nephropathy and acid-base imbalances, which may be compounded by amphotericin B toxicity and post-COVID renal damage [31, 32].

To mitigate these adverse effects, liposomal amphotericin B has been increasingly utilized as a preferable alternative in recent years due to its reduced nephrotoxic potential. In addition, maintaining hemodynamic stability in the perioperative phase is essential for renal damage mitigation and anesthetic management of the patients. Moreover, preoperative anxiolytic pharmacotherapy is essential for effective perioperative anesthetic management in patients [33].

In a retrospective study, researchers primarily examined the effects of preoperatively administered midazolam on hemodynamically unstable patients [11]. In the aforementioned study, desflurane was used as the inhalational anaesthetic agent. Various studies have declared elevated serum creatinine levels and thrombocytopenia as the most significant predictors of mortality in individuals with mucormycosis [34]. In contrast to these findings, a retrospective study reported that two deceased patients exhibited thrombocytopenia but had normal serum creatinine levels [11].

Literature review confirmed that surgical debridement of necrotic tissues in patients with rhinocerebral mucormycosis significantly reduces mortality [35-37]. In contrast, mortality rates reach 70% in patients treated with antifungal therapy alone, whereas this rate drops to 14% in those who undergo surgery combined with antifungal therapy [4]. Moreover, studies have revealed that the concurrent presence of absolute neutropenia, diffuse sepsis, immunosuppression, and multiorgan failure exacerbates morbidity and mortality in mucormycosis patients [36, 38].

### Anesthetic challenges in the management of rhino-orbital cerebral mucormycosis

In patients with ROCM, surgical debridement may involve various procedures, including functional endoscopic sinus surgery, orbital exenteration, enucleation, mandibulectomy, maxillectomy, and craniotomy [16] (Figure 1). These cases present numerous perioperative challenges. Preoperative assessment frequently identifies systemic complications related to post-COVID-19 sequelae, such as pulmonary, cardiovascular, renal, and coagulation dysfunction [39]. Given that these patients present clinical manifestations, such as dyspnea, persistent cough, fatigue, and reduced exercise capacity, arterial blood gas analysis, chest X-ray, and peripheral oxygen saturation monitoring are critical.

According to a previous study, 59% of patients exhibited peripheral oxygen saturation below 90% on room air, while nearly 86% of them displayed lung involvement across all four zones on chest X-ray. Moreover, preoperative arterial blood gas analysis indicated that 34% of patients had a partial pressure of oxygen between 60–80 mmHg, whereas 7% of patients presented with severe hypoxemia (PaO_2_ <60 mmHg). For the management of mucormycosis, amphotericin B was initiated at a dosage of 1 mg/kg, administered as a 4–6-hour infusion, and continued for 2–5 days before surgery [16]. It should be mentioned that 80% of these patients presented with hypokalemia, hypovolemia, and fever. Laboratory findings revealed that 55% of patients exhibited blood sugar levels between 160-180 mg/dL, while 47% demonstrated hyperglycemia exceeding 200 mg/dL on the operative day. These findings underscore the critical importance of rigorous perioperative glucose monitoring. In the aforementioned study, four patients had renal dysfunction, which required postoperative dialysis. Moreover, COVID-19 in these patients may lead to cardiac complications, including primary manifestations (e.g., arrhythmias, myocardial infarction, and myocarditis) or secondary effects (e.g., myocardial injury with elevated biomarkers and heart failure) [40].

These effects can result in dysrhythmias, ischemic events, and nonischemic cardiomyopathy. Consequently, continuous electrocardiographic monitoring is essential. Additionally, preoperative echocardiographic assessment is recommended. Patients with ROCM should receive anticoagulant therapy, such as oral rivaroxaban or low-molecular-weight heparin, promptly after recovering from COVID-19 [41, 42]. Studies have shown that approximately 12% of these patients have hemoglobin levels below 8 g/dL, raising concerns about potential blood loss in this population [16].

In addition, the interval between COVID-19 infection and surgical intervention is a critical consideration. Current recommendations suggest delaying elective procedures for at least 4–6 weeks following COVID-19 infection to minimize perioperative risks [43]. However, in emergency cases, surgery may be performed within two weeks of achieving a COVID-negative status. For anesthetic induction, propofol is the preferred agent due to its favorable profile in patients with potential airway hyperreactivity and anticipated difficult intubation. Regarding neuromuscular blockade, short-acting depolarizing agents, such as suxamethonium, are recommended for endotracheal intubation, while atracurium is preferred for the maintenance of muscle relaxation, particularly in patients with renal impairment. Additionally, total intravenous anesthesia presents a viable alternative for functional endoscopic sinus surgery, offering advantages in certain clinical scenarios. Bhalerao et al. in their study (2022) recommended the use of inhalational anesthetic isoflurane for maintenance of anesthesia, as they observed that continuous infusion could lead to delayed recovery in critically ill patients [16]. They assessed mucormycosis patients and found that approximately 43% exhibited Mallampati grades 3 and 4, along with an inter-incisor distance of less than 2.5 cm [16]. Restricted mouth opening was also observed, attributed to pain, joint erosion, and stiffness associated with diabetes mellitus. To facilitate endotracheal intubation, a large Macintosh laryngoscope blade or a C-Mac video laryngoscope (Karl Storz GmbH & Co., Germany) was employed, often with a stylet to guide the endotracheal tube. In cases of palatal perforation, a gauze pack was utilized to improve laryngoscopic visualization. Moreover, loose teeth posed a risk and required careful handling during the insertion of laryngoscopy.

Postoperative extubation presented challenges due to factors, such as delayed recovery, electrolyte and acid-base disturbances, hypothermia, compromised pulmonary function, central nervous system involvement, and nasal packing following maxillectomy or palatal resection. Given that both functional endoscopic sinus surgery and endotracheal intubation are aerosol-generating procedures, adherence to personal protective equipment protocols was emphasized [44]. Tuncer et al. (2024) reported that patients with mucormycosis often demonstrated compromised hemodynamic stability during surgery, necessitating inotropic support and invasive arterial monitoring [45]. Given the frequent presence of comorbidities and the aggressive nature of the infection, postoperative ICU management is critical in these cases [46].

## Anesthetic management of rhino-orbital cerebral mucormycosis

### Diabetic and hypertensive patient

A 62-year-old male presented to the ear, nose, and throat (ENT) outpatient department with primary symptoms of bilateral ptosis, limited ocular motility persisting for two weeks, and left mandibular swelling accompanied by pain for one week. The patient had contracted COVID-19 seven days after receiving the COVID-19 vaccination, with infection confirmed via polymerase chain reaction (PCR). During his illness, he was treated with beta-lactam antibiotics, intravenous hydrocortisone, and subcutaneous enoxaparin. His oxygen saturation remained stable at ≥ 93% on room air, with no signs of respiratory distress. Laboratory investigations revealed elevated HbA1c (9.4%) and a random blood glucose (RBS) level of 514 mg/dL. Electrocardiography indicated left axis deviation. Chest X-ray demonstrated peripheral opacities along with bilateral basal lung infiltrates. Magnetic resonance imaging (MRI) of the brain revealed left sinonasal-orbital-cerebral mucormycosis extending to the hard palate, accompanied by cerebral venous thrombosis and a temporal lobe abscess. The patient was initiated on amphotericin B therapy for antifungal treatment. This patient had pre-existing diabetes mellitus and hypertension.

Perioperative hyperglycemia presents significant challenges, adversely affecting both anesthetic management and surgical outcomes. Additionally, post-COVID pulmonary compromise increases the risk of general anesthesia-induced lung injury, potentially prolonging hospitalization. Poor glycemic control elevates the risk of aspiration due to diabetic gastroparesis, necessitating rapid-sequence intubation. Given the anticipated difficult airway and reduced cardiopulmonary reserve from post-COVID sequelae, airway management strategies included the presence of a senior anesthesiologist, preoxygenation, and video laryngoscopy (Table 1).

**Table 1:** Summary of key anaesthetic challenges and management strategies in rhino-orbital-cerebral mucormycosis.

Challenge	Pathophysiology	Management Strategies
Difficult airway	Fungal debris, supraglottic edema, trismus, palatal perforation	• Video laryngoscopy as first-line
		• Fiberoptic intubation in case of severe obstruction
		• Avoidance of the nasal route
		• Emergency tracheostomy kit available
Hemodynamic instability	Amphotericin B toxicity (hypotension), sepsis, and COVID-19 cardiac sequelae	• Invasive arterial pressure monitoring
		• Vasopressor support (norepinephrine)
		• CVP-guided fluid resuscitation
Renal dysfunction	Amphotericin B nephrotoxicity, diabetic nephropathy, and COVID-19 AKI	• Liposomal amphotericin B formulation
		• Strict electrolyte monitoring (K^+^, Mg^2+^)
		• Avoidance of nephrotoxic anesthetics
Metabolic Derangements	Uncontrolled diabetes, steroid use, and amphotericin-induced hypokalemia	• Intraoperative insulin infusion protocol
		• Frequent glucose monitoring (target 140-180 mg/dL)
		• K^+^ replacement
Thromboembolic risk	COVID-19 hypercoagulability, vascular invasion by fungi	• LMWH prophylaxis post-op
		• Intraoperative pneumatic compression
		• Monitor D-dimer test

CVP: Central venous pressure, AKI: Acute Kidney Injury, LMWH: Low-Molecular-Weight Heparin

Due to labile blood pressure secondary to autonomic dysfunction, preoperative preparation required vasopressors, inotropes, and antihypertensive medications. The patient’s uncontrolled hypertension and left axis deviation on electrocardiography warranted further evaluation with a two-dimensional echocardiogram; however, the emergent nature of the surgery limited preoperative optimization. Judicious fluid therapy and advanced hemodynamic monitoring were crucial in enhancing perioperative outcomes. Hypotension was strictly avoided to prevent acute kidney injury and exacerbation of post-COVID ventilation-perfusion (V/Q) mismatch. A lung-protective ventilation strategy was employed to minimize barotrauma and volutrauma in the already compromised lungs. Intraoperative hypertension increased bleeding risk, necessitating a careful balance between transfusion-related acute lung injury and the consequences of anemia-induced reduced oxygen delivery. Continuous temperature monitoring was maintained to prevent hypothermia and subsequent lactic acidosis. Proper patient positioning was crucial to avoid nerve compression and pressure sores. Given the high metabolic stress, glucose monitoring was essential to avert diabetic ketoacidosis or hyperosmolar hyperglycemic state. Additionally, due to amphotericin B therapy, meticulous electrolyte and fluid management was prioritized [47].

### Perioperative considerations in a patient with multiple comorbidities

A 43-year-old female presented to the ENT outpatient department with a one-week history of restricted ocular motility and blurred vision, accompanied by a 1.5-month history of facial swelling and toothache, as well as unilateral hearing loss persisting for one month. Diagnostic evaluation revealed uncontrolled diabetes mellitus, with an elevated HbA1c of 9%. Laboratory investigations demonstrated anemia (hemoglobin: 9 mg/dL), with peripheral blood smear findings consistent with macrocytosis, suggestive of pernicious anemia. Electrocardiography and chest X-ray findings were unremarkable. The patient had a five-year history of hypothyroidism secondary to Hashimoto’s thyroiditis, confirmed by elevated anti-thyroid peroxidase antibodies, and was maintained on levothyroxine (50 mcg daily).

However, recent thyroid function tests indicated poor control, with a thyroid-stimulating hormone (TSH) level of 26 mIU/L. The MRI of the brain revealed invasive fungal sinusitis with extensive involvement of the infraorbital, premaxillary, palatal, and infratemporal regions. The patient was initiated on oral posaconazole (300 mg daily) as antifungal therapy. Surgical debridement of the affected palatal and sinonasal tissues was performed under general anesthesia [47]. This patient presented with a complex medical history, including uncontrolled diabetes mellitus, Hashimoto's thyroiditis, and pernicious anemia. The immunomodulatory effects of COVID-19 infection potentially exacerbated the underlying autoimmune conditions, as evidenced by disease flare-ups following viral infection. Preoperative assessment revealed significant hypothyroidism (TSH 26 mIU/L), indicating suboptimal thyroid replacement therapy. The coexistence of pernicious anemia necessitated a comprehensive evaluation for other potential autoimmune disorders, such as rheumatoid arthritis, which could further complicate airway management.

Administration of general anesthesia in this patient presented multiple challenges due to four significant, poorly controlled comorbidities. Particular attention was required regarding drug dosing, as hypothyroidism may delay drug metabolism and clearance. Additionally, the inherent bleeding tendency associated with hypothyroidism mandated meticulous surgical technique and consideration of hemostatic agents. Differential diagnosis of postoperative coma in this patient would be particularly complex, requiring distinction between diabetic ketoacidosis, myxedema coma, and prolonged anesthetic effects. Due to the presence of pernicious anemia, nitrous oxide was contraindicated to avoid potential hematological complications. The immunocompromised state associated with hypothyroidism, characterized by lymphocyte dysfunction, necessitated strict adherence to aseptic protocols throughout the perioperative period [48, 49].

### A complex case of invasive fungal sinusitis

A 48-year-old female presented to the ophthalmology outpatient department with a 40-day history of visual disturbances (blurred and double vision) accompanied by a persistent headache. The patient reported a recent episode of oral ulceration treated with a tapering course of oral prednisolone (60 mg initial dose followed by 30 mg for six days).

Notably, the medical history was negative for fever, myalgia, malignancy, or other immunosuppressive therapies. The patient had a 13-year history of poorly controlled type 2 diabetes mellitus and hypertension, maintained on pharmacotherapy but with inconsistent clinical follow-up. Laboratory investigations revealed significant metabolic derangement, including markedly elevated HbA1c (13.4%), RBS of 330 mg/dL, anemia (hemoglobin 8.9 g/dL), and elevated inflammatory markers (neutrophil-to-lymphocyte ratio >9).

While COVID-19 reverse transcription PCR (RT-PCR) testing was negative, inflammatory biomarkers were substantially elevated, including the D-dimer: 933 ng/mL, Interleukin-6: 111 pg/mL, and C-reactive protein: 39 mg/L. Neuroimaging demonstrated extensive bilateral sinus involvement (maxillary, sphenoid, ethmoid) with left frontal sinusitis complicated by orbital and intracranial extension. In light of these findings, antifungal therapy with liposomal amphotericin B (275 mg) was initiated. Given the disease progression and orbital compromise, the patient underwent emergent surgical management consisting of sinus debridement and left eye evisceration [47].

This case presented a clinically intriguing scenario of an asymptomatic COVID-19 patient whose initial presentation consisted solely of oral ulceration, subsequently treated with steroid therapy. Subsequent serological evaluation revealed elevated anti-SARS-CoV-2 IgM antibodies, indicating recent infection despite no history of vaccination. This finding suggests both a missed COVID-19 diagnosis and potential overzealous steroid administration, which may have predisposed to opportunistic fungal infection. The longstanding poorly controlled diabetes mellitus and hypertension of the patient (previously discussed implications) compounded the clinical complexity.

The elevated neutrophil-to-lymphocyte ratio of > 9 correlated with severe COVID-19 progression as documented in literature [50]. Markedly increased inflammatory biomarkers confirmed an active cytokine storm state, characterized by substantial elevation in oxygen demand and concomitant reduction in pulmonary compliance due to inflammatory lung injury. These pathophysiological changes significantly increased the anesthetic risk during general anesthesia. Notably, the patient demonstrated significantly elevated D-dimer levels without prior anticoagulation therapy. This necessitated intraoperative mechanical and pneumatic thromboprophylaxis measures and immediate postoperative initiation of pharmacological thromboprophylaxis (following surgical consultation regarding bleeding risks).

Intraoperative management challenges included elevated airway pressures, requiring implementation of lung-protective ventilation strategies. The clinical dilemma of managing ongoing cytokine storm (where corticosteroids remain the only evidence-based treatment) against the potential exacerbation of fungal infection required careful therapeutic consideration.

In another case, a 61-year-old female presented to the ENT outpatient department with primary symptoms of cough, left periorbital swelling, and blurred vision [47]. She reported a 15-day history of high-grade fever and dyspnea, with a confirmed COVID-19 diagnosis via RT-PCR. Laboratory investigations revealed markedly elevated COVID-19 biomarkers. High-resolution computed tomography on day 9 demonstrated ground-glass opacities, with a CT severity score of 12/25. Hematological analysis indicated leukopenia (white blood cell count: 1030/μl), a neutrophil-to-lymphocyte ratio of 3, and hemoglobin levels of 9.3 mg/dl, while platelet counts remained within the normal range. Chest X-ray exhibited bilateral pulmonary opacification, more pronounced on the right side (3 mmol/dl). Magnetic resonance imaging identified left maxillary sinusitis with palatal erosion. The patient had a three-year history of poorly controlled diabetes mellitus, with irregular medication adherence .

Therapeutic management included supplemental oxygen via a face mask at 7 L/min to maintain oxygen saturation above 95%, along with systemic antibiotics (intravenous ceftriaxone 1 g and doxycycline 100 mg for 7 days) and inhaled side. Moreover, the arterial blood gas analysis revealed hypoxemia (PaO_2_: 68 mmHg) with elevated lactate fludrocortisone (400 mcg, four puffs twice daily). Given the clinical suspicion of fungal infection, liposomal amphotericin B (150 mg) was initiated, and surgical debridement under general anesthesia was planned. A potential contributing factor in this case was the deposition of inhaled corticosteroids in the nasal and oral mucosa, which might have created a favourable environment for Mucorales colonization and subsequent invasive fungal growth.

## Conclusion

Management of ROCM presented formidable anesthetic challenges, necessitating a multidisciplinary approach to address its rapid progression and high mortality. This review underscored the critical perioperative considerations, including difficult airway management due to fungal invasion and edema, hemodynamic instability exacerbated by amphotericin B toxicity, and the imperative of glycemic control in diabetic patients. The integration of advanced monitoring techniques, tailored anesthetic protocols, and meticulous preoperative optimization is essential to mitigate risks. Case studies highlight the compounding effects of post-COVID-19 sequelae, uncontrolled comorbidities, and immunosuppression, further complicating care. Key recommendations include the use of video laryngoscopy for airway security, avoidance of the internal jugular vein for central access, and liposomal amphotericin B to reduce nephrotoxicity.

Surgical debridement combined with antifungal therapy remains the cornerstone of treatment, significantly improving survival rates compared to medical management alone. Future research should focus on standardizing anesthetic guidelines and the exploration of novel antifungals with fewer adverse effects. By addressing these challenges through evidence-based strategies, anesthesiologists can enhance perioperative outcomes in this high-risk population, reducing morbidity and mortality in ROCM patients.
